# Outcomes of Mechanically Ventilated Patients With Nosocomial Tracheobronchitis

**DOI:** 10.7759/cureus.20259

**Published:** 2021-12-08

**Authors:** Feroz Ali Khan, Usman M Qazi, Shakeeb Ahmad J Durrani, Ayesha Saleem, Anum Masroor, Kiran Abbas

**Affiliations:** 1 Department of Orthopaedic Surgery, Maqsood Medical Complex General Hospital, Peshawar, PAK; 2 Department of Community Medicine, Khyber Medical College, Peshawar, PAK; 3 Department of Medicine, Shaheed Farid Khan District Headquarter Hospital, Hangu, PAK; 4 Department of General Surgery, Hayatabad Medical Complex, Peshawar, PAK; 5 Department of Psychiatry, California Institute of Behavioral Neurosciences & Psychology, Fairfield, USA; 6 Department of Psychiatry, Psychiatric Care Associates, Englewood, USA; 7 Department of Medicine, Khyber Medical College, Peshawar, PAK; 8 Department of Medicine, Jinnah Postgraduate Medical Centre, Karachi, PAK

**Keywords:** ventilation, patient outcomes, mechanical ventilation, ntb, nosocomial tracheobronchitis

## Abstract

Introduction

Ventilator-associated tracheobronchitis is a condition that occurs prior to the development of ventilator-associated pneumonia among patients who have been intubated. This study aimed to determine the impact of nosocomial tracheobronchitis (NTB) related to new bacteria on the outcome in patients with chronic obstructive pulmonary disease (COPD).

Methodology

A prospective, observational study was conducted in the department of surgical ICU of a tertiary care hospital between May 2019 and December 2019. All patients ventilated, irrespective of age and gender, were enrolled in the study. Individuals who had nosocomial pneumonia, before or followed by NTB, were excluded. Throughout the study, endotracheal aspirates for quantitative bacterial cultures were obtained routinely on admission, weekly thereafter, and whenever NTB or nosocomial pneumonia was suspected. All data were prospectively collected by the researchers from admission day till discharge or death of the patient. The outcomes of NTB patients were compared with those without NTB.

Results

A total of 24 patients with NTB and 214 patients without NTB were evaluated. There were a total of 24 patients who were diagnosed with NTB and 214 patients were NTB negative. There was a dominance of the male gender in the NTB group; however, the difference was not significant. The most common complication in patients was renal failure. During hospitalization, the *Aspergillus* tracheobronchitis (ATB) rate was significantly higher in patients with NTB as compared to patients with no NTB, i.e., 18 (75%) vs. 80 (37.4%) (p < 0.001). The mean length of stay in patients with NTB was significantly greater than the NTB negative group (p < 0.0001). The mortality in the case group was significantly greater than in the NTB negative group, i.e., 66.67% vs. 48.50% (p = 0.03).

Conclusion

NTB is associated with an increased duration of mechanical ventilation and hospitalization in intensive care units. The mortality rate in the NTB group was considerably higher than in the patients who did not have NTB. Future studies can explore the interventional and management aspect of the disease, such as determining whether early administration of broad-spectrum antibiotics can help improve the prognostic outcome of patients with NTB on mechanical ventilation.

## Introduction

Ventilator-associated tracheobronchitis is a condition that occurs prior to the development of ventilator-associated pneumonia among patients who have been intubated. Hospital-acquired tracheobronchitis is common among seriously ill patients, having an incidence of 2.7-10% [1-4]. A vast multicenter study was conducted in Italy in 2008 to evaluate the epidemiology of infections acquired in the ICU [5]. The study involved 9,493 patients that were admitted into 71 adult ICUs, out of which 11.4% suffered from ICU-acquired infection.

Mechanical ventilation through the orotracheal or nasotracheal tube was associated with a higher risk of nosocomial lower respiratory tract infection (NLRTI), as the tubes can evade natural defenses of the body, allowing for bacterial leakage and secretions around the cuff, and damage the ciliated epithelium of the trachea, thereby reducing bacterial clearance [6]. The endotracheal biofilm acts as a reservoir for pathogens, and fragments of the film may pass into the lungs causing transmission of pathogens into the airways by evading the host’s immune response [7]. Though multiple studies have been conducted to evaluate the risk factors and prognosis of nosocomial pneumonia, little has been done to study nosocomial tracheobronchitis (NTB) [8-10].

According to the authors, the effects of NTB on patient outcomes have never been assessed. However, it has been established that the condition is associated with higher rates of morbidity and mortality [10]. Additionally, it can result in prolonged ICU admission and mechanical ventilation. This can cause weaning difficulties and nosocomial pneumonia in intubated patients. Thus, a prospective study was conducted to assess the incidence and causative pathogens that play a role in the outcome of NTB.

## Materials and methods

A prospective, observational study was conducted in the department of surgical ICU of a tertiary care hospital between May 2019 and December 2019. Ethical approval was obtained from the institutional review board of Maqsood Medical Complex General Hospital prior to the study (reference: MMC/IRB/E-4548). A non-probability convenience sampling technique was used to recruit participants in the study. All patients ventilated, irrespective of age and gender, were enrolled in the study. Individuals who had nosocomial pneumonia, before or followed by NTB, were excluded.

Throughout the study, endotracheal aspirates for quantitative bacterial cultures were obtained routinely on admission, weekly thereafter, and whenever NTB or nosocomial pneumonia was suspected. All data were prospectively collected by the researchers from admission day till discharge or death of the patient. NTB was identified by prospective surveillance of nosocomial infections. Tracheobronchitis was defined by all the following criteria: (1) fever (>38°C) with no other recognizable cause; (2) new or increased sputum production; (3) positive (≥106 cfu/ml) endotracheal aspirate culture yielding new bacteria; and (4) no radiographic evidence of nosocomial pneumonia [7].

Mechanical ventilation was defined as any period of mechanical ventilation with tracheal intubation. Multidrug-resistant bacteria were defined as methicillin-resistant *Staphylococcus aureus*, ceftazidime- or imipenem-resistant *Pseudomonas aeruginosa*, *Acinetobacter baumannii*, extending-spectrum ß-lactamase-producing gram-negative bacilli, and *Stenotrophomonas maltophilia*. Outcomes evaluated included ICU mortality, duration of mechanical ventilation, and ICU stay. The outcomes of NTB patients were compared with those without NTB.

The analysis was performed via Statistical Package for Social Sciences (SPSS) version 26 (IBM SPSS Statistics, Armonk, NY). For continuous variables such as age and hospital stay, mean with standard deviation were calculated. For categorical variables including gender, rate of NTB, etc., frequency and percentage were determined. Using chi-square and Student's t-test, associations between NTB and clinical or sociodemographic variables were explored. Differences between patients were considered significant if the p-value was <0.05.

## Results

A total of 24 patients with NTB and 214 patients without NTB were evaluated. There were a total of 24 patients who were diagnosed with NTB and 214 patients were NTB negative. There was a dominance of the male gender in the NTB group; however, the difference was not significant. The most common complication in patients was renal failure. During hospitalization, the *Aspergillus* tracheobronchitis (ATB) rate was significantly higher in patients with NTB as compared to patients with no NTB, i.e., 18 (75%) vs. 80 (37.4%) (p < 0.001) (Table 1). Out of the 24 patients with NTB, 14 (58.3%) were ≥60 years of age while out of the 214 patients without NTB, 150 (70.1%) were ≥60 years of age.

**Table 1:** Main characteristics of study patients (NTB vs. no NTB). NTB: nosocomial tracheobronchitis; SAPS: simplified acute physiology score; COPD: chronic obstructive pulmonary disease.

Characteristics	NTB positive	NTB negative	p-value
Aged ≥60 years	14 (58.3%)	150 (70.1%)	0.238
Male gender	17 (72.2%)	138 (64.6%)	0.536
Secondary hospitalization	23 (95.8%)	213 (99.5%)	0.06
SAPS II	38.6 ± 20.6	35.4 ± 16.6	0.234
Diabetes mellitus	4 (16.7%)	28 (13.1%)	0.626
COPD	9 (37.5%)	54 (25.2%)	0.196
Organ failure			
Respiratory	16 (66.7%)	130 (60.7%)	0.572
Cardiac	3 (12.5%)	44 (20.6%)	0.347
Neurologic	1 (4.2%)	23 (10.7%)	0.31
Digestive	3 (12.5%)	27 (12.6%)	0.987
Renal	7 (29.2%)	54 (25.2%)	0.676

The mean length of stay in patients with NTB was significantly greater than the NTB negative group (p < 0.0001). The mortality in the case group was significantly greater than in the NTB negative group, i.e., 66.67% vs. 48.50% (p = 0.03) (Table 2).

**Table 2:** Outcomes of mechanically ventilated patients with NTB. NTB: nosocomial tracheobronchitis.

Outcome variables	NTB positive		NTB negative		p-value
	Frequency/mean	Percentage/SD	Frequency/mean	Percentage/SD	
Mean ICU length of stay (in days)	44.6	18.9	45.6	15.5	0.678
Mean length of mechanical ventilation (in days)	36.7	12.5	20.8	5.6	<0.0001
Mortality					
Yes	17	66.67%	104	48.50%	0.03
No	7	33.33%	110	51.40%	

## Discussion

In our study, we found that the mean length of stay (p < 0.678) and the mean length of mechanical ventilation (p < 0.0001) were greater in the NTB group than in the NTB negative group. The mortality rate in the NTB group (66.67%) was higher than in the NTB negative group (48.50%) (p = 0.03). Ventilator-associated tracheobronchitis (VAT) is a condition that usually affects patients that are on mechanical ventilation [11]. Many studies suggest that VAT is linked to a longer duration of mechanical ventilation and longer stay in ICU [12,13].

A study conducted by Nseir et al. found NTB to be linked with longer ICU stay and length of mechanical ventilation in patients [14]. The rate of mortality, however, was similar in patients with NTB in absence of nosocomial pneumonia in contrast to patients who did not have NTB. Treatment of NTB with antimicrobials led to a better prognosis. Similarly, Ray et al. found VAT in 13.2% of the population, and most of these patients were found to have neurological issues (58%) [15]. The average time leading to VAT post mechanical ventilation was almost seven days and post ICU was 10 days. In this study, *Pseudomonas aeruginosa* and *Acinetobacter* species were the most commonly isolated organisms leading to VAT.

Furthermore, a study by Migiyama et al. looked at the risk factors leading to high *Pseudomonas aeruginosa* density in mechanically ventilated patients [16]. Hyperglycemia was found as a risk factor for high *Pseudomonas aeruginosa* density along with prolonged mechanical ventilation greater than 28 days and respiratory disorders that were diagnosed when admitted to the ICU. Alves et al. found that using antibiotic therapy does not improve the prognosis of VAT as it does not reduce the risk of mechanical ventilation, mortality, and hospitalization but reduces progression to pneumonia due to ventilator [17]. Moreover, Hashemi et al. diagnosed VAT on the basis of fever, presence of mucus and tracheal secretions, and no lung involvement [18]. Out of the 69 patients, 23 patients (33.3%) developed VAP and the remaining 38 died (55%), leading to high ICU mortality. The organisms *Pseudomonas aeruginosa*, *Enterobacter*, and *Acinetobacter baumannii* were some of the common bacterial isolates. This was similar to our study as the NTB group was associated with longer ICU stay and mortality. Similarly, another study by Lee and Saydain also identified VAT as a major risk factor for VAP in patients with lowered immunity and bacterial colonization of the oral cavity [19]. The study had some limitations. For instance, a small number of NTB positive cases limited the interference of the findings to a larger population. The current study acts as a catalyst for future studies, which can explore the risk factors of NTB in a more diversified study population.

## Conclusions

NTB is associated with an increased duration of mechanical ventilation and hospitalization in intensive care units. The mortality rate in the NTB group was considerably higher than in the patients who did not have NTB. Our study assessed the clinical profile of patients and associated factors with NTB. Future studies can explore the interventional and management aspect of the disease such as determining whether early administration of broad-spectrum antibiotics can help improve the prognostic outcome of patients with NTB on mechanical ventilation.

## References

[1] Agrafiotis M, Siempos II, Falagas ME (2010). Frequency, prevention, outcome and treatment of ventilator-associated tracheobronchitis: systematic review and meta-analysis. Respir Med.

[2] Kampf G, Wischnewski N, Schulgen G, Schumacher M, Daschner F (1998). Prevalence and risk factors for nosocomial lower respiratory tract infections in German hospitals. J Clin Epidemiol.

[3] Rello J, Ausina V, Castella J, Net A, Prats G (1992). Nosocomial respiratory tract infections in multiple trauma patients. Influence of level of consciousness with implications for therapy. Chest.

[4] Craven DE, Hjalmarson KI (2010). Ventilator-associated tracheobronchitis and pneumonia: thinking outside the box. Clin Infect Dis.

[5] Malacarne P, Langer M, Nascimben E, Moro ML, Giudici D, Lampati L, Bertolini G (2008). Building a continuous multicenter infection surveillance system in the intensive care unit: findings from the initial data set of 9,493 patients from 71 Italian intensive care units. Crit Care Med.

[6] Kofteridis DP, Papadakis JA, Bouros D (2004). Nosocomial lower respiratory tract infections: prevalence and risk factors in 14 Greek hospitals. Eur J Clin Microbiol Infect Dis.

[7] Madalina Mihai M, Maria Holban A, Giurcaneanu C (2015). Microbial biofilms: impact on the pathogenesis of periodontitis, cystic fibrosis, chronic wounds and medical device-related infections. Curr Top Med Chem.

[8] Zaragoza R, Vidal-Cortés P, Aguilar G (2020). Update of the treatment of nosocomial pneumonia in the ICU. Crit Care.

[9] NanZhu Y, Xin L, Xianghua Y, Jun C, Min L (2019). Risk factors analysis of nosocomial pneumonia in elderly patients with acute cerebral infraction. Medicine (Baltimore).

[10] Chastre J, Trouillet JL, Vuagnat A, Joly-Guillou ML (1996). Nosocomial pneumonia caused by Acinetobacter spp. Acinetobacter.

[11] Koulenti D, Arvaniti K, Judd M (2020). Ventilator-associated tracheobronchitis: to treat or not to treat?. Antibiotics (Basel).

[12] Moreau AS, Martin-Loeches I, Povoa P (2018). Impact of immunosuppression on incidence, aetiology and outcome of ventilator-associated lower respiratory tract infections. Eur Respir J.

[13] Heimes E, Baier M, Forstner C (2020). Effect of antiviral therapy on the outcome of mechanically ventilated patients with herpes simplex virus type 1 in BAL fluid: a retrospective cohort study. Chest.

[14] Nseir S, Di Pompeo C, Pronnier P (2002). Nosocomial tracheobronchitis in mechanically ventilated patients: incidence, aetiology and outcome. Eur Respir J.

[15] Ray U, Ramasubban S, Chakravarty C, Goswami L, Dutta S (2017). A prospective study of ventilator-associated tracheobronchitis: incidence and etiology in intensive care unit of a tertiary care hospital. Lung India.

[16] Migiyama Y, Sakata S, Iyama S (2021). Airway Pseudomonas aeruginosa density in mechanically ventilated patients: clinical impact and relation to therapeutic efficacy of antibiotics. Crit Care.

[17] Alves AE, Pereira JM (2018). Antibiotic therapy in ventilator-associated tracheobronchitis: a literature review. Rev Bras Ter Intensiva.

[18] Hashemi SH, Hashemi N, Esna-Ashari F, Taher A, Dehghan A (2017). Clinical features and antimicrobial resistance of bacterial agents of ventilator-associated tracheobronchitis in Hamedan, Iran. Oman Med J.

[19] Lee SJ, Saydain G (2017). Ventilator associated tracheobronchitis: a call for more evidence. Lung India.

